# HIV-related symptoms and management in HIV and antiretroviral therapy patients in KwaZulu-Natal, South Africa: A longitudinal study

**DOI:** 10.1080/17290376.2013.870119

**Published:** 2014-01-03

**Authors:** Karl Peltzer

**Affiliations:** a PhD, Research Director, Research Programme HIV/AIDS, STI, and TB (HAST), Human Sciences Research Council, South Africa; b Research Associate with the University of Limpopo, South Africa; c Visiting professor at the ASEAN Institute for Health Development, Mahidol University, Thailand

**Keywords:** HIV symptoms, management strategies, antiretroviral therapy, longitudinal study, symptômes du VIH, stratégies de gestion, thérapie antirétrovirale, étude longitudinale

## Abstract

**Aim:**

The study aimed to determine the prevalence, predictors, and self-reported management of HIV- or ARV-related symptoms among HIV patients prior to antiretroviral therapy (ART) and over three time points while receiving ART in KwaZulu-Natal, South Africa.

**Method:**

A total of 735 consecutive patients (29.8% male and 70.2% female) who attended three HIV clinics completed assessments prior to ARV initiation, 519 after 6 months, 557 after 12 months, and 499 after 20 months on ART.

**Results:**

The HIV patients reported an average of 7.5 symptoms (prior to ART), 1.2 symptoms after 6 months on ART, 0.3 symptoms after 12 months on ART, and 0.2 symptoms after 20 months on ART on the day of the interview, with a higher symptom frequency amongst patients who were not employed, had lower CD4 cell counts, experienced internalised stigma, and used alcohol. The most common symptoms or conditions identified by the self-report included tuberculosis, diarrhoea, headaches, rash, nausea and vomiting, pain, neuropathy, lack of appetite, cough, and chills. Overall, the participants reported medications as the most frequently occurring management strategy, with the second being spiritual, and the third being complementary or traditional treatments. The use of all other management strategies decreased over the four different assessment periods from prior to ART to 20 months on ART.

**Conclusion:**

This study found a high symptom burden among HIV patients, which significantly decreased with progression on antiretroviral treatment. Several symptoms that persisted over time and several sociodemographic factors were identified that can guide symptom management. The utilisation of different symptom management strategies (medical, spiritual, complementary, and traditional) should be taken into consideration in HIV treatment.

## Abstract (FR)

**Objectif:**

L'étude visait à déterminer la prévalence, les prédicteurs et la gestion, déclarée par les patients, des symptômes liés au VIH ou aux médicaments antirétroviraux avant la thérapie antirétrovirale (TAR) et à trois périodes déterminées au cours de la TAR. L'étude a eu lieu à KwaZulu-Natal, en Afrique du Sud.

**Méthode:**

Des patients ont fait l'objet d'évaluations dans trois cliniques du VIH. Voici le nombre de patients qui ont réalisé les évaluations au cours des différentes étapes: 735 patients (29,8% homme, 70,2% femme) avant le lancement de la TAR, 519 patients après 6 mois de traitement, 557 après 12 mois, et 499 après 20 mois.

**Résultats:**

Au moment de l'évaluation, les patients ont déclaré le nombre de symptômes suivant, en moyenne: 7,5 symptômes avant la TAR, 1,2 symptôme après 6 mois de TAR, 0,3 symptôme après 12 mois de TAR et 0,2 symptôme après 20 mois de TAR. La fréquence des symptômes était plus élevée parmi les patients sans emploi, ayant une faible numération des lymphocytes T CD4, qui ont fait l'objet de stigmate intériorisée, et qui consomment de l'alcool. Selon les déclarations des participants, les symptômes ou les états les plus courants comprennent notamment la tuberculose, la diarrhée, les maux de tête, les éruptions cutanées, la nausée, le vomissement et les douleurs, la neuropathie, la perte d'appétit, la toux, et les frissons. En général, les participants ont déclaré que les médicaments constituent la principale stratégie de gestion des symptômes, suivi de l'approche spirituelle. Les traitements complémentaires ou traditionnels arrivent au troisième rang. Toutes les stratégies de gestion ont connu une baisse au cours des quatre peériodes d'évaluation, soit d'avant la TAR jusqu'à après 20 mois de TAR.

**Conclusion:**

Au cours de cette étude, il a été possible de conclure que les patients atteints de VIH doivent composer avec beaucoup de symptômes, et les symptômes diminuent au cours du traitement antirétroviral. Plusieurs symptomes persistants au fil du temps et plusieurs facteurs sociodémographiques ont été cernés. Ces facteurs pourront servir à aiguiller la gestion des symptômes. L'utilisation de différentes stratégies de gestion des symptômes (médicale, spirituelle, complémentaire et traditionnelle) doit être prise en considération dans le cadre d'un traitement contre le VIH.

## Introduction

Few studies, especially longitudinal, have described the self-reported symptoms of people living with HIV (PLHIV) and antiretroviral therapy (ART) patients in sub-Saharan Africa (Bhengu, Ncama, McInerney, Wantland, Nicholas, Corless, *et al.* 2011; Friend-du Preez & Peltzer 2010; Makoae, Seboni, Molosiwa, Moleko, Human, Sukati, *et al.* 2005; Peltzer & Phaswana-Mafuya 2008; Wakeham, Harding, Bamukama, Levin, Kissa, Parkes-Ratanshi, *et al.* 2010), resulting in a paucity of evidence-based research on which to design appropriate interventions. ‘HIV/AIDS symptoms can result from the disease itself, from secondary complications of the disease, or from side effects of highly active ART (HAART) and other medications related to comorbidities. HIV symptoms are the single most important indicators for patients and practitioners. Symptoms prompt patients to seek medical attention and provide health care providers with essential clues about changes in health status and quality of life’ (Portillo, Holzemer & Chou 2007: 1824). This study describes the symptom experiences of PLHIV about to commence ART and at three time points over 20 months on ART who were residing in resource-poor communities in KwaZulu-Natal, South Africa. The relationship between current symptoms and management strategies in this setting is also investigated.

It is estimated that approximately 5.6 million people are living with HIV and AIDS and 1.7 million were on ART in South Africa in 2012, more than in any other country (UNAIDS 2012). The highest prevalence (24.7% in the general population) is found in the province of KwaZulu-Natal (National Department of Health 2012). Rollout of ART began in 2004 and the number of people enrolled (1.7 million) is now the highest in the world (UNAIDS 2012). Although as people begin treatment, they can expect to live longer, most patients initiate HAART at advanced stages of the disease with very low CD4 counts and in poor health (May, Boulle, Phiri, Messou, Myer, Wood, *et al.* 2010). In the Southern African context and in other studies, a higher symptom frequency has been reported by those in advanced stages of the disease (patients who have recently been hospitalised, been given an AIDS diagnosis, and those with lower CD4 counts), patients of lower socio-economic status (Gonzalez, Penedo, Llabre, Durán, Antoni, Schneiderman, *et al.* 2007; Harding, Lampe, Norwood, Date, Clucas, Fisher, *et al.* 2010; Makoae *et al.* 2005; Peltzer & Phaswana-Mafuya 2008), those on ART (Harding, Molloy, Easterbrook, Frame & Higginson 2006), and those with a poor ART adherence (Gonzalez *et al.* 2007; Harding *et al.* 2010).

The study aimed to determine prevalence, predictors, and self-reported management of HIV- or ARV-related symptoms among HIV patients prior to ART and over three time points while receiving ART in KwaZulu-Natal, South Africa.

## Methods

### Sampling and procedure

This is a prospective study of all treatment-naxve patients (*N* = 735) recruited from the three public hospitals in the Uthukela health district in KwaZulu-Natal from October 2007 to February 2008. All ARV-naxve patients who were about to commence ARVs (18 years and above) and who consecutively attended the HIV clinics during the recruitment period were eligible for this study. Systematic sampling was used by asking health care providers for referrals of ART-naive patients (eligible for ARV treatment but who had not commenced ARV treatment yet). Physicians from the three selected public clinics asked every consecutively visiting ART-naive patient meeting the inclusion criteria of being 18 years or over if they would like to complete a confidential survey and interview concerning their health and social situation. This would include information from their medical records on details of their medical condition, laboratory tests, and treatment. It was made clear to patients that their participation in this study was voluntary and that a decision not to participate would not affect their medical care. If the potential participant indicated an interest in participating, the health care provider then referred them to an external Human Sciences Research Council research assistant. The ART-naïve patients were then asked to sign and complete a consent form before the interview took place in a private area in or outside the clinic. The interviews were conducted by four trained external HSRC researchers (one or two per HIV clinic) in interviewer-administered semi-structured interviews. Permission to access patient medical records was sought from both the patient and the health worker/manager. Questionnaires were anonymised, with no personal identifying information recorded on them. Recruitment took place over a period of four months, with a 97.8% participation rate. Data were collected using an interviewer-administered semi-structured questionnaire.

The questionnaire was translated into the major language spoken in the study area (Zulu) and verified by a second translator. Where inconsistencies were found, these were corrected (Peltzer, Friend-du Preez, Ramlagan & Fomundam 2008). The patients were then interviewed again at the 6, 12, and 20 months clinic visits post-initiation of ARV. Patients who did not attend the planned follow-up were contacted by telephone and up to two home visits before being considered lost to follow-up. Sample size calculations using Epi Info Version 7.1 found that based on the expected frequency of patients who would have symptoms of 25% (based on Makoae *et al.* 2005, confidence limit 5%, three clusters of study, a confident level of 99% yielded a cluster size of 166), a total of 498 was chosen as the minimum sample size. The study protocol was approved by the Human Sciences Research Council ethics committee, the KwaZulu-Natal Department of Health, the Uthukela Health District and the three superintendents of the three study hospitals.

### Measures

The patients were interviewed with an anonymous questionnaire that requests information on sociodemographic characteristics, clinical history, and health-related characteristics and health beliefs. Clinical data relating to date of HIV diagnosis, HIV acquisition and transmission risk factors, current CD4 cell count, and viral load were obtained from the medical chart.

#### The revised signs and symptoms checklist for persons with HIV disease

The SSC-HIVrev is a 72-item checklist of HIV/AIDS-specific physical and psychological symptoms scored using the following scale: 0 = not checked (not present today), 1 = mild, 2 = moderate, and 3 = severe (Holzemer, Hudson, Kirksey, Hamilton & Bakken 2001). The validity and reliability of the instrument have previously been reported for a US sample (Holzemer *et al.* 2001) and various African countries (Makoae *et al.* 2005), including South Africa (Peltzer & Phaswana-Mafuya 2008); the reliability estimates ranged from 0.76 to 0.94. The Cronbach *α* reliability coefficient of this 64-item scale was 0.95 at baseline and 0.84, 0.95, and 0.78 for the three subsequent assessment periods, indicating the excellent internal consistency reliability of the items. The presence of any symptom was added up to form the total HIV-related symptom burden.

### Self-reported HIV-related symptoms and management

Participants were asked to describe three to six HIV- and/or ARV-related symptoms they had experienced, list the strategies (medications, complementary or traditional treatments, self-comforting, changing diet, seeking help, exercise, spiritual care, and daily thoughts/activities to make themselves feel better) they used to manage these symptoms, and rate the perceived effectiveness of these strategies (Sukati, Mndebele, Makoa, Ramukumba, Makoae, Seboni, *et al.* 2005).

### Internalised AIDS stigma

We used the six-item internalised AIDS-related stigma scale for people infected with HIV (Kalichman, Simbayi, Cloete, Mthembu, Mkhonta & Ginindza 2009b). Items reflected self-defacing beliefs and negative perceptions of PLHIV/AIDS. For example, ‘It is difficult to tell other people about my HIV infection’. Response options ranged from 1 = strongly agree to 4 = strongly disagree. The Cronbach *α* reliability coefficient of this six-item scale was 0.80 at baseline and 0.64, 0.66, and 0.78 for the three subsequent assessment periods, indicating the excellent to moderate internal consistency reliability of the items.

### HIV/AIDS discrimination experiences

To assess AIDS-related discrimination, we asked participants whether they had experienced seven discrimination-related events, e.g. whether they had been treated differently since they had disclosed their HIV status to friends and family; whether being HIV positive had caused them to lose a job or a place to stay; and whether they had experienced discrimination because they are HIV positive (Simbayi, Kalichman, Strebel, Cloete, Henda & Nqeketo 2007). Response options were ‘yes’ or ‘no’. Cronbach's alpha for this sample was 0.54.

### Adherence assessment

The 30-day visual analogue scale (VAS) provided an overall adherence assessment for a longer time interval. The VAS is a valid method of assessing medication adherence (Kalichman, Amaral, Swetzes, Jones, Macy, Kalichman, *et al.* 2009a) and has been validated in resource-limited settings (e.g. Maneesriwongul, Tulathong, Fennie & Williams 2006). Adherence was calculated as the % of doses taken over those prescribed. Adherence levels assessed from the VAS are defined as follows: full adherence = 100%, partial adherence ≥ 95% and < 100%, and non-adherence < 95% of prescribed doses taken since the last refill.

### Alcohol use disorder

The alcohol use disorders identification test (AUDIT-C) focuses solely upon the consumption of alcohol (i.e. the frequency of drinking, the quantity consumed at a typical occasion, and the frequency of heavy episodic drinking (i.e. consumption of six standard drinks or more on a single occasion – in South Africa, a standard drink is 12 g alcohol)) (Babor, Higgins-Biddle, Saunders & Monteiro 2001). Because AUDIT-C is reported to be less sensitive at identifying risk drinking in women, the cut-off points for binge drinking for women were reduced by one unit compared with men (Freeborn, Polen, Hollis & Senft 2000). Gual, Segura, Contel, Heather and Colon (2002) recommend a cut-off point of ≥ 5 for men and ≥ 4 for women; despite this, the false positive rate was 46.5% among male and 63.3% among female patients when compared with a clinical diagnosis of risky drinking. Cronbach's alpha for the AUDIT-C in this sample was 0.91 at Time 4.

### Data analysis

Data were analysed using Statistical Package for the Social Sciences for Windows software application programme version 17.0. Frequencies, means, standard deviations, median, and interquartile ranges were calculated to describe the sample. To identify the pattern of factors characterising the HIV-related symptom burden at any assessment, linear regression models based on generalised estimating equations (GEEs) were used. These models allowed for the consideration of the correlation among within-subject repeated measures (Twisk 1997). For the univariate analyses, simple linear regression was used to identify factors associated with the HIV-related symptom burden; variables with *p*-values lower than 0.05 were entered in the corresponding multiple regression model. A forward procedure based on the quasi likelihood ratio test produced the final model.

## Results

A total of 735 patients (217 men and 518 women) completed a baseline questionnaire at Time 1 prior to initiating ART. Follow-up questionnaires were completed at the 6-month follow-up by 519 patients within this cohort (139 men and 370 women) who had now been on ART for 6 months, 12 months later by 557 patients within this cohort (157 men and 396 women), and 20 months later by 499 patients (126 men and 333 women) and 236 (32.1%) participants were lost to follow-up (including transfers): 83 (11.3%) were known to have died, 74 (10.1%) transferred care elsewhere, 14 (1.9%) refused participation, 12 (1.6%) were not initiated on ART, and 53 (7.2%) could not be traced. At Time 4, the HIV medications for 380 (76.3%) patients were Lamivudine (3TC), Stavudine (d4T) + Efavirenz and for 118 (23.7%) patients were Lamivudine (3TC), Stavudine (d4T) + Nevirapine.

### Sample characteristics

The mean age of the participants at baseline assessment was 35.9 years (SD = 9.7) and the educational level of the majority (81.0%) was less than Grade 12. Almost three-quarters (71.8%) had never married, 61.2% were unemployed and 20.2% employed, more than half (52.3%) had a child care grant, 29.9% had a formal salary, and 7.9% had no income. Almost two-thirds (63.3%) resided in a rural area, most (73.5%) had recently been (within the past year) diagnosed as being HIV positive, and almost all (96.1%) had disclosed their HIV status to someone. While baseline characteristics were similar between men and women, women were more likely to be younger and receiving social (including child care) grants and men were more likely to be married or cohabiting, employed, and have a formal salary. The median CD4 count at the 20-month follow-up was 446 cells/cu.mm compared with 261 cells/cu.mm at the 12-month follow-up, 130 cells/cu.mm at the 6-month follow-up, and 119 cells/cu.mm prior to ARV initiation. A study attrition analysis comparing participants who left the study with those who stayed found significant differences in terms of a lower educational level, rural residence, employment status (which is most likely a function of increased mobility for more educated individuals who can find work in urban areas), and lower HIV symptoms for those who stayed in the study and no significant differences in terms of gender, age, religion, income, time since HIV diagnosis, CD4 cell counts, internalised stigma, discrimination experience, and alcohol use (see Table 1).

**Table 1.** Sample characteristics and study attrition analysis

	Baseline (Time 1)		Stayed in the study (Time 4)			
Variable	*N* = 735 or *M*	%or SD	*N* = 499	%or SD	*χ*^2^ or *t*-test	*p*
*Sex*						
Male	217	29.5	137	63.1	3.2	0.07
Female	518	70.5	362	69.9		
*Age, range 18–67*	35.9	9.7	36.1	9.5	−0.82	0.42
*Education*						
Grade 7 or less	279	38.1	199	71.3	4.39	0.04
Grade 8–11	314	42.9	214	68.2	0.02	0.9
Grade 12 or more	139	19	84	60.4	4.39	0.04
*Religion*						
African or none	187	25.4	121	64.7	1.17	0.28
Mainstream Christian	154	21	99	64.3	1.16	0.28
Charismatic	271	36.9	194	71.6	2.69	0.1
*Residence*						
Rural (village)	338	46.2	215	63.6	5.19	0.02
Rural (farm)	125	17.1	95	76	4.59	0.03
Urban (informal settlements)	41	5.6	32	78	2.07	0.15
Urban (formal settlements)	227	31.1	154	67.8	0	1
*Employment status*						
Housewife/houseman	99	13.5	74	74.7	2.37	0.12
Unemployed	448	61.2	290	64.7	5.78	0.02
Employed	148	20.2	110	74.3	3.38	0.07
Pensioner/disabled/student	24	3.4	14	56	1.72	0.19
*Income*						
Formal salary	215	29.9	157	73	2.94	0.09
Family member contributions	133	18.5	89	66.9	0.18	0.67
Social grants	264	36.7	182	68.9	0.04	0.84
Disability grant (for chronic illness)	129	23.8	89	69	0.27	0.61
Child care support grant	383	52.3	262	68.4	0.13	0.71
No income	57	7.9	38	63.2	0.81	0.37
*Time since HIV diagnosis*						
≤1 year (2007/8)	540	73.5	367	68	0.01	0.95
1–2 years (2006)	73	9.9	55	75.3	2.06	0.15
>2 years (2005–1995)	122	16.6	77	63.1	1.53	0.22
*CD4 count (cells/µL)*						
1–99	106	19.7	91	85.8	0.2	0.66
100–349	345	64.2	300	87	0.03	0.86
≥350	86	16	77	89.5	0.52	0.47
*The number of HIV symptoms (range: 0–64)*	7.2	9.5	6.8	9.3	2.64	0.008
*Internalised stigma (range: 0–6)*	3.8	2.4	4.2	2.7	−0.99	0.32
*Discrimination experiences (range: 0–7)*	0.3	1	0.3	0.6	0.5	6.7
*AUDIT scores (range: 0– 12)*	0.4	1.4	0.6	1.3	−0.78	0.43

### HIV-related symptom changes over 20 months of follow-up

The baseline mean total score on the HIV-symptom scale for the 735 patients was 7.5 (SD = 9.6), which significantly decreased at Time 2–1.2 (SD = 2.6), at Time 3–0.3 (SD = 2.1), and at Time 4–0.2 (SD = 0.9) (*F* = 2193.69; *p* < 0.001). An examination of individual HIV-symptom items indicated that the percentage of patients scoring mild, moderate, or severe (defined as a score of 1 or more) decreased over time for all items. The most obvious change was for ‘concerned over weight loss’ and ‘dry mouth'; approximately one-third (35.7% and 31.7%) of patients scored ‘yes’ on these items at baseline, but none scored ‘yes’ at the 20-month follow-up. The five most frequently reported symptoms at Time 1 were concern over weight loss, headaches, dry mouth, memory loss, and weakness; at Time 2, chills, headaches, diarrhoea, concern over weight gain, and dry mouth; at Time 3, headaches, diarrhoea, numbness/tingling of legs, lack of appetite, and painful joints; and at Time 4, painful joints, diarrhoea, lack of appetite, fever, and headaches. The scores for each HIV-symptom item at each of the four assessments are given in Table 2.

**Table 2.** Symptom frequency (mild, moderate, or severe) in percentage over time.

Problem	Time 1	Rank	Time 2	Rank	Time 3	Rank	Time 4	Rank
1. Concern over weight loss	35.7	1	1.5	29	0.4	22	0	
2. Headaches	32.1	2	6.3	2	2.5	1	1.2	5
3. Dry mouth	31.7	3	4.0	5	0.5	12	0	
4. Memory loss	27.0	4	3.8	6	0.4	23	0	
5. Weakness	24.8	5	3.4	8	0.5	13	0	
6. Thirsty	24.2	6	2.9	14	0		0	
7. Painful joints	23.6	7	3.8	7	1.3	5	1.8	1
8. Chills (feeling very cold)	23.1	8	9.9	1	0.9	8	0.2	15
9. Chest pain	22.9	9	3.1	11	1.3	6	0.2	16
10. Numbness/tingling of legs	21.3	10	3.1	12	1.4	3	0	
11. Lack of appetite	20.9	11	2.7	6	1.4	4	1.4	3
12. Diarrhoea	20.3	12	5.4	3	2.0	2	1.6	2
13. Numbness/tingling of feet/toes	20.3	13	2.3	21	0.4	24	0	
14. Heart racing	17.5	14	1.1	35	0.2	41	0	
15. Fatigue	17.4	15	1.9	24	0.2	42	0	
16. White spots in mouth/thrush	17.3	16	1.3	30	0.5	14	0.4	11
17. Numbness/tingling of hands/fingers	17.1	17	2.9	15	0.4	25	0	
18. Night sweats	16.3	18	2.3	22	0		0.2	17
19. Dizziness	15.6	19	3.4	9	0.4	26	1.2	6
20. Muscle aches	15.0	20	1.3	31	0.4	27	0.2	18
21. Depression (sadness)	14.8	21	2.5	19	0.5	15	0	
22. Fear/worry	14.2	22	2.5	20	0.4	28	0	
23. Nausea	14.1	23	2.7	17	0.4	29	0	
24. Rash	13.8	24	2.7	18	0.5	16	0.4	12
25. Numbness/tingling of arms	13.1	25	1.7	27	0		0	
26. Day sweats	12.8	26	2.1	23	0.2	43	0	
27. Difficulty concentrating	12.0	27	1.1	36	0.4	30	0	
28. Abdominal pain	11.9	28	1.3	32	0.5	17	0.8	9
29. Insomnia/cannot sleep	11.6	29	0.6	49	0		0	
30. Hump on back of neck/shoulders	11.6	30	1.1	37	0.4	31	0	
31. Vomiting	11.5	31	1.0	41	0.7	11	0	
32. Loose stools	10.9	32	1.9	25	0.4	32	0.2	19
33. Coughing/problems catching breath	10.5	33	0.8	43	0.9	9	1.2	7
34. Fever	10.0	34	3.4	10	1.1	7	1.4	4
35. Easy bruising	9.4	35	0.4	54	0		0.2	20
36. Itchy skin	9.3	36	1.3	33	0.9	10	0.6	10
37. Concern over weight gain	8.6	37	4.6	4	0.5	18	0	
38. Anxious	8.5	38	0.4	55	0.2	44	0	
39. Burning with urination	8.3	39	0.8	44	0.4	33	0.2	21
40. Shortness of breath at rest	7.9	40	0.4	56	0	55	0	
41. Shortness of breath with activity	7.4	41	0.6	50	0.2	45	0	
42. Weight gain in stomach area	7.4	42	1.7	28	0.5	19	0.2	22
43. Swollen feet	7.0	43	1.1	38	0.4	34	0.4	13
44. Skinny arms and legs	6.7	44	0.8	45	0.2	46	0	
45. Rectal discharge	6.7	45	1.1	39	0.4	35	0	
46. Mouth ulcers	6.6	46	0.2	61	0.2	47	0	
47. Wheezing	6.0	47	0.6	51	0.2	48	0	
48. Seizures/tremors	6.0	48	1.1	40	0		0	
49. Constipation	5.8	49	0.2	62	0.4	36	0.2	23
50. Blurred vision	5.7	50	2.1	24	0.4	37	0	
51. Sore throat	5.6	51	0.2	63	0.5	20	0.2	24
52. Rectal itching	5.5	52	0.8	46	0		0	
53. Gas/bloating	5.2	53	3.1	13	0.5	21	0	
54. Painful swallowing	5.2	54	0	64	0.4	38	0	
55. Nipple discharge	5.2	55	0.8	47	0		0.2	25
56. Rectal bleeding	4.9	56	1.3	35	0		0	
57. Swollen glands	4.8	57	0.4	57	0		1.2	8
58. Prominent leg veins	4.4	58	1.0	42	0.4	39	0	
59. Flushing	4.2	59	0.4	58	0		0.4	14
60. Sore/bleeding gums	3.8	60	0.8	48	0		0	
61. Breast pain/changes	3.7	61	0.4	59	0.4	40	0.2	26
62. Sores or lumps on genitals	3.7	62	0.6	52	0		0.2	27
63. Blood in spit/sputum	3.4	63	0.4	60	0.2	49	0.2	28
64. Nose bleeds	2.6	64	0.6	53	0		0.2	29
Total mean (SD)	7.5 (9.6)		1.2 (2.6)		0.3 (2.1)		0.2 (0.9)	

### Predictors of HIV-related symptom burden

To identify at any assessment the pattern of factors characterising the HIV-related symptom burden (all symptoms together), linear regression models based on generalised estimating equations (GEEs) were used. When univariate analyses were employed and in the GEE multiple regression model (see Table 3), not being employed, lower CD4 cell counts, internalised stigma, and alcohol use were associated with a greater HIV-related symptom burden. Furthermore, in a separate model, ART adherence (from Times 2 to 4) was not found to be associated with the HIV-related symptom burden (from Times 2 to 4) (*β* coefficient 0.06 (95% CI: –0.04 to 0.201) (*p* = 0.091).

**Table 3.** Predictors of the HIV-related symptom burden.

Variables	*β* coefficient (95% CI)	*p*	Adjusted *β* coefficient (95% CI)^a^	*p*
*Sociodemographic variables*				
Age	0.01 (–0.004, 0.014)	0.307	–	
Male vs. female	0.05 (–0.14, 0.25)	0.589	–	
Educational level	0.05 (0.01, 0.09)	0.014	0.12 (–0.01,0.24)	0.05
Never married/widowed/separated/divorced vs. married or cohabiting				
	0.05 (–0.14, –0.25)	0.602	–	
Urban vs. rural residence	−0.77 (–0.95, –0.59)	<0.001	0.24 (–0.33, 0.81)	0.406
Formal/informal employment vs. not	−1.03 (–1.23, –0.84)	<0.001	−2.36 (–3.00, –1.72)	<0.001
*Clinical variables*				
Time since HIV diagnosis	−0.09 (–0.21, 0.03)	0.147	–	
CD4 count (cells/µL)	−3.41 (–5.44, –1.39)	0.001	−0.01 (–0.011, –0.017)	<0.001
*Social variables*				
Internalised stigma scores	0.54 (0.49, 0.59)	<0.001	0.66 (0.57, 0.76)	<0.001
Discrimination experiences score	−0.31 (–0.44, –0.17)	<0.001	0.25 (–0.14, 0.64)	0.204
Alcohol use score	0.24 (0.07, 0.42)	0.007	0.33 (0.14,0.51)	<0.001

*Note:* All variables with *p* < .05 in the baseline HIV-related symptom burden adjusted model were eligible for the multivariate model.

a Goodness of fit quasi-likelihood under independence model criterion value = 44,318.46.

### HIV- or ARV-related self-reported symptoms and management

Participants first listed 3–6 HIV- or ARV-related symptoms. The five most common symptoms or conditions identified at Time 1 (prior to ART) were tuberculosis, headaches, diarrhoea, weight loss, and cough; at Time 2, headaches, diarrhoea, rash, nausea and vomiting, and pains; at Time 3, headaches, numbness/tingling, pains, rash, and lack of appetite; and at Time 4, diarrhoea, rash, headaches, chills, and pains (see Table 4).

**Table 4.** HIV- or ARV-related self-reported symptoms.

	Time 1		Time 2		Time 3		Time 4	
	*N*	Rank order	*N*	Rank order	*N*	Rank order	*N*	Rank order
Headaches	56	2	83	1	38	1	8	3
Diarrhoea	48	3	51	2	17	3	97	1
Rash	25	8	45	3	15	4	10	2
Nausea and vomiting	25	8	41	4	11	6	1	9
Numbness/tingling of legs/arms/hands/feet (neuropathy)	17	13	21	5	23	2	1	9
Pain (chest, leg, body)	24	9	20	6	17	3	5	5
Lack of appetite	26	7	11	7	13	5	4	6
Fatigue	22	11	6	9	2	10	1	9
Tuberculosis	175	1			1	11	2	8
Herpes	26	38	2					
Stomach problem	19	12	5	10	7	7	3	7
Thrush	17	13	5	10				
Chills (feeling very cold)					5	8	6	4
Weight loss	39	4						
Cough	33	5	9	8				
Discharge disorder; STI	28	6	2	12	3	9		
Memory loss			9	8	7	7		
Night sweats	5	16	5	10				
Itchy skin	7	14	2	12				
Swollen glands	6	15						
Dry mouth			3	11	1	11		
Fever	4	17						
Sexual problem	1	19			1	11		
Weight gain					1	11		

Regarding management strategies for the self-reported HIV- or ARV-related symptoms, medications were the most frequently mentioned care strategies and ranked first in overall use, ranging from 22.5% at Time 3 to 85.9% at Time 2. This was followed by spiritual care at 33.3% at Time 1 and at 46.4% at Time 2 and complementary or traditional treatments at 37% at Time 1 and at 30.9% at Time 2. Overall, all different management strategies decreased over the four different assessment periods from prior to ART to 20 months on ART (see Table 5).

**Table 5.** Type of symptom management strategy and perceived effectiveness.

	Time 1 (*n* = 735)		Time 2 (*n* = 519)		Time 3 (*n* = 557)		Time 4 (*n* = 499)	
	*N* (%)	*M* effect	*N* (%)	*M* effect	*N* (%)	*M* effect	*N* (%)	*M* effect
Medication	403 (65.7)	1.9 (0.9)	177 (85.9)	2.4 (0.6)	100 (22.5)	2.2 (0.6)	39 (28.9)	2.8 (0.4)
Complementary/traditional treatments	230 (37.0)	1.5 (0.6)	25 (30.9)	2.4 (0.6)	13 (2.9)	2.8 (0.5)	0	0
Self-comforting	250 (39.9)	1.5 (0.7)	23 (28.4)	1.5 (0.6)	13 (2.9)	2.2 (0.7)	4 (3.0)	2.3 (0.5)
Change in diet	377 (39.7)	1.5 (0.7)	17 (21.8)	1.8 (0.4)	4 (0.9)	2.0 (0.8)	1 (0.7)	3
Seeking help	241 (38.5)	1.5 (0.7)	17 (22.7)	1.4 (0.5)	8 (1.8)	1.8 (0.7)	2 (1.5)	2.5 (0.7)
Exercise	37 (5.9)	1.9 (0.9)	5 (7.4)	1.7 (0.8)	4 (0.9)	2.8 (0.5)	0	0
Spiritual care	208 (33.3)	1.6 (0.7)	45 (46.4)	2.2 (0.7)	20 (4.5)	2.6 (0.6)	2 (1.5)	2.0 (0.0)
Daily thoughts/activities	233 (37.3)	1.4 (0.6)	21 (26.6)	1.3 (0.5)	5 (1.1)	2.0 (1.0)	0	0

## Discussion

In this study, HIV patients reported an average of 7.5 symptoms (prior to ART), 1.2 symptoms after 6 months on ART, 0.3 symptoms after 12 months on ART, and 0.2 symptoms after 20 months on ART on the day of the interview, with a higher symptom frequency amongst patients who were not employed, experienced internalised stigma, used alcohol, and had lower CD4 cell counts. The duration of HIV diagnosis was not associated with the HIV-related symptom burden. Other studies (Gonzalez et al. 2007; Makoae *et al.* 2005) also found that lower CD4 cell counts were associated with higher HIV-related symptoms. As found in other studies (Gonzalez *et al.* 2007; Harding *et al.* 2010; Makoae *et al.* 2005; Peltzer & Phaswana-Mafuya 2008), this study found that lower socio-economic status or not being employed was associated with the HIV-related symptom burden. This study did not find that poor ART adherence was associated with HIV-related symptoms, which was also found in a study in KwaZulu-Natal, South Africa (Bhengu *et al.* 2011), but some other studies (Gonzalez *et al.* 2007; Harding *et al.* 2010) found an association between poor ART adherence and HIV-related symptoms.

The five most frequently reported symptoms on the structured questionnaire at Time 1 were concern over weight loss, headaches, dry mouth, memory loss, and weakness; at Time 2, chills, headaches, diarrhoea, concern over weight gain, and dry mouth; at Time 3, headaches, diarrhoea, numbness/tingling of the legs, lack of appetite, and painful joints; and at Time 4, painful joints, diarrhoea, lack of appetite, fever, and headaches. In an open-ended question, the most common symptoms or conditions identified included tuberculosis, diarrhoea, headaches, rash, nausea and vomiting, and pains. Similar symptoms have been reported from other studies in Southern Africa (Bhengu *et al.* 2011; Makoae *et al.* 2005; Peltzer & Phaswana-Mafuya 2008). It is interesting to note that diarrhoea and pain, including headache and numbness/neuropathy, were frequently mentioned across the different assessment periods in this study, as also found in other studies (Bhengu *et al.* 2011; Sukati *et al.* 2005; Tsai, Hsiung& Holzemer 2002). This suggests that the management of pain and peripheral neuropathy should be prioritised by clinicians providing HIV/AIDS care (Bhengu *et al.* 2011; Nicholas, Voss, Wantland, Lindgren, Huang, Holzemer, *et al.* 2010). Harding, Simms, Alexander, Collins, Combo and Memiah (2013) showed the evidence that integrated HIV outpatient palliative care in the presence of ART can ameliorate the high burden of pain, symptoms, and other multidimensional problems that persist alongside HIV treatment.

Overall, the participants reported medications as the most frequently occurring management strategy and the most effective, second was spiritual care, and third was complementary or traditional treatments. This finding suggests that participants in this study used their medical provider as a major source of the management of their symptoms. Furthermore, spiritual, complementary, or traditional treatments apparently play an important role in PLHIV. All types of management strategies decreased over the four different assessment periods from prior to ART to 20 months on ART. In a different study in Southern Africa using the same management categories, medications were also ranked first, complementary treatments were ranked second, and spiritual care was ranked seventh (Sukati *et al.* 2005). In terms of traditional and complementary treatments in this sample, the use of herbal therapies for HIV declined significantly from 36.6% prior to ARV therapy to 8.0% after 6 months, 4.1% after 12 months, and 0.6% after 20 months on ARVs. Faith healing methods (including spiritual practices and prayer) declined from 35.8% to 22.1%, 20.8%, and 15.5%, respectively (Peltzer, Friend-du Preez, Ramlagan, Fomundam, Anderson & Chanetsa 2011). The use of different symptom management strategies should be taken into consideration in HIV treatment.

## Limitations

Viral load data were only available for a few participants and so this category was therefore excluded from the analysis. Furthermore, the assessment of ART adherence and other measures was relied on the self-report. The study results may be biased in favour of those who survived and were healthy enough to participate in follow-up. Sample attrition is a methodological artefact that can potentially influence longitudinal studies (Burgoyne, Rourke, Behrens & Salit 2004). The symptoms described by participants may represent symptoms of HIV disease, side effects of antiretroviral treatment, or a combination of both effects of medications and HIV disease (Bhengu *et al.* 2011). Finally, the findings are derived from a sample of men and women residing in one district in one province in South Africa. Thus, caution is urged in generalising the findings to other districts and provinces in the country.

## Conclusions and implications

This study found a high symptom burden among HIV patients, which significantly decreased with progression on antiretroviral treatment. This means that antiretroviral treatment is effective in reducing the HIV-related symptom burden among HIV patients.

Several symptoms that persisted over time and several sociodemographic factors that were identified can guide symptom management using the existing WHO clinical guidance that emphasises the essential role of palliative care alongside HIV treatment.

The utilisation of different symptom management strategies (medical, spiritual, complementary, and traditional) should be taken into consideration in HIV treatment.
